# Bisphenol AF Induced Neurodevelopmental Toxicity of Human Neural Progenitor Cells via Nrf2/HO-1 Pathway

**DOI:** 10.3390/ijms26125685

**Published:** 2025-06-13

**Authors:** Huan Luo, Mengchao Ying, Yun Yang, Qian Huo, Xinyu Hong, Gonghua Tao, Ping Xiao

**Affiliations:** 1 Shanghai Municipal Center for Disease Control & Prevention, Shanghai 201107, China; 2 State Environmental Protection Key Laboratory of Environmental Health Impact Assessment of Emerging Contaminants, Shanghai 200233, China

**Keywords:** BPAF, ReNcell CX cells, neurodevelopmental toxicity, Nrf2/HO-1 pathway

## Abstract

Bisphenol AF (BPAF) is widely utilized as an analog of bisphenol A (BPA) in the plastics industry. However, there is limited evidence on its neurodevelopmental toxicity. Existing studies suggest that BPAF has greater accumulation in vivo than other bisphenol analogs, and could pass through the placental barrier and the blood–brain barrier. In this study, we used the human neural progenitor cells line ReNcell CX, which was derived from 14-week human cortical brain tissue, as an in vitro model to investigate the neurodevelopmental toxicity effects of BPAF and BPA on ReNcell CX cells, and explored the possible mechanism by which BPAF induced neurodevelopmental toxicity on ReNcell CX cells. The results showed that BPAF reduced the proliferation of neural progenitor cells and changed the differentiation towards neurons after exposure for 24 h. Compared with BPA, ReNcell CX cells are more susceptible to BPAF exposure. In a 3D neurospheres model, BPAF affected the distance that neurons migrated outwards at the concentration of 2 μM. Furthermore, BPAF increased ROS levels in cells and reduced the expression of key proteins in the Nrf2/HO-1 pathway and its downstream molecules, such as SOD, GSH, and CAT. In conclusion, BPAF induces damage to critical nodes in neural progenitor cell development through the Nrf2/HO-1 pathway. Therefore, clarifying its neurodevelopmental toxicity and elaborating on the neurodevelopmental toxicity effects and mechanisms of bisphenol AF will help identify intervention targets for neurodevelopmental toxicity, and will have important public health significance for the safety assessment and risk prediction of bisphenol-related chemicals.

## 1. Introduction

Bisphenol A (BPA) and its alternatives are used in a variety of industrial manufacturing processes. Bisphenol AF (BPAF), as a fluorine-containing derivative of BPA, has also been widely used as a food contact polymer, fluoroelastomer, and electronic material in recent years [1]. Some studies have suggested that BPAF can interact more easily with estrogen receptors than other bisphenols and also might be more prone to bioaccumulation [2]. The persistent contamination refractoriness of BPAF has raised concerns about potential adverse effects. The prevalence of nervous system-related diseases such as learning disabilities, cognitive impairment, and depression caused by impaired neurodevelopment are increasing gradually, which is closely associated with exposure to chemical pollution in the environment [3]. However, the exact impact of BPAF on nervous system development remains uncertain.

Strict and orderly neurogenesis is crucial for the development of a normal nervous system. This process includes the complete process of self-renewal of neural progenitor cells, asymmetric division to form neural progenitor cells, the formation of various functional cells such as neurons and glial cells, and the migration of cells to specific areas to establish synaptic connections [4]. Disturbance of this process may result in a reduction in the number of neurons and cortical dysfunction [5]. Studies have demonstrated that exposure to BPAF in paternal zebrafish can reduce the hatchability of F1 larvae and shorten the body length of offspring [6,7], exerting adverse effects on neurogenesis in the central nervous system. It can also reduce the learning and memory ability of zebrafish and influence the expression of nervous system-related genes. Perinatal exposure to BPAF may cause cognitive dysfunction in adult offspring mice [8]. Multiple in vitro studies have indicated that BPAF may shorten and reduce the length and number of neurites of neurons and induce neuronal apoptosis [9]. However, the specific mechanism is still unclear.

The previous literature has affirmed that neurodevelopmental toxicity is closely associated with the oxidative stress induced by reactive oxygen species (ROS) [10]. The normal level of intracellular ROS is a key factor affecting the physiological proliferation and differentiation of neural progenitor cells. At high concentrations, ROS have adverse effects on cell components, such as DNA, proteins, and lipids [11]. Studies have demonstrated that BPAF might induce oxidative stress in cells, cause intracellular ROS increase, interfere with various signal transduction pathways in cells, and affect cell growth, proliferation, and apoptosis [12,13]. The nuclear factor erythrocyte 2-related factor 2 (Nrf2) is a significant antioxidant transcription factor in regulating the expression of antioxidant proteins, such as heme oxygenase 1 (HO-1). Under physiological circumstances, Nrf2 binds to the cysteine protein Kelch-like ECH-associated protein 1 (Keap1), which is located in the cytoplasm [14]. The Nrf2 signaling pathway can regulate the self-renewal, proliferation, and differentiation of stem cells by controlling the redox status of stem cells [15]. The intracellular ROS level is a key factor affecting the proliferation and differentiation of neural progenitor cells. Studies have shown that transient activation of the Nrf2 signaling pathway can promote the self-renewal, proliferation, and differentiation of stem cells by resisting oxidative stress. However, exposure to continuous oxidative stress can lead to the apoptosis or premature aging of stem cells and damage neurogenesis [16]. PI3K/AKT is a signaling pathway associated with cell proliferation, differentiation, and apoptosis. Phosphatase and tensin homolog (PTEN) can regulate the dephosphorylation of phosphatidylinositol triphosphate (PIP3) to phosphatidylinositol diphosphate, thereby determining whether the PI3K/AKT signaling pathway is activated. The activation of the protein kinase B (AKT) signaling pathway can facilitate the dissociation of Nrf2 and Keap1, thereby regulating the expression of Nrf2 in the cell nucleus [17]. Studies have indicated that cognitive impairment and neurological impairment can be alleviated by activating the PI3K/AKT signaling pathway and promoting Nrf2 transcription [18,19]. Unfortunately, the role of the AKT/Nrf2/HO-1 pathway in BPAF-induced neurodevelopmental toxic effects remains unclear.

ReNcell CX cells cultured in vitro have the ability of self-renewal, multi-directional differentiation, and hold unique advantages in the assessment of neurodevelopmental toxicity [20]. They can simulate the development process of the human nervous system to conduct in-depth research on the mechanism of toxicity. The ReNcell CX cells can also reduce the number of experimental animals for studies. Consequently, this new model may be more sensitive and reliable for predicting the harm of chemicals to humans, providing a broad space for studying neurodevelopmental toxicity.

To sum up, this study used ReNcell CX cells cultured as an in vitro model, and utilized toxicity indicators and surface markers of neural stem cell proliferation and damage as detection endpoints to observe the adverse effects of BPAF on the neurogenesis of ReNcell CX cells and the role of the AKT/Nrf2/HO-1 pathway in the BPAF-induced neurodevelopmental toxicity of ReNcell CX cells. The implementation of this project can provide not only a scientific basis for safety assessment and risk management of BPAF, but also ideas for the promotion and application of neurotoxicity screening and prediction technology on the basis of in vitro stem cell models.

## 2. Results

### 2.1. BPAF Reduced the Cell Activity and Proliferation of ReNcell CX Cells to a Greater Extent than BPA

**Figure 1:**
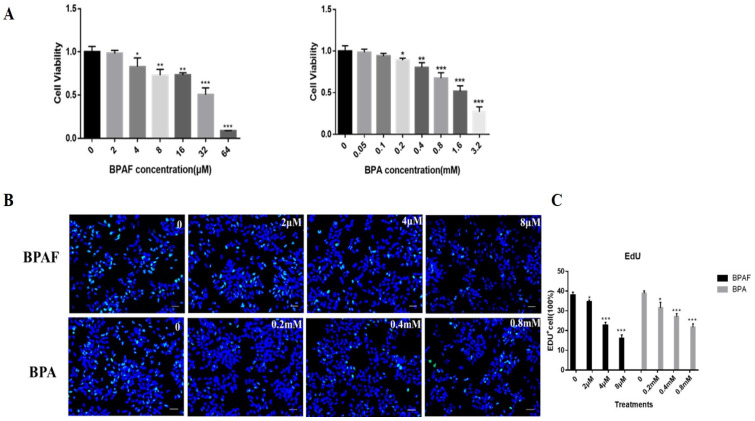
BPAF induced cytotoxicity and decreased the number of EdU+ (5-ethynyl-2′-deoxyuridine) cells. (**A**) The cytotoxicity was tested by CCK8 assay kit. (**B**) The cell proliferation was shown by the number of EdU+ cells (Green), and cell nuclei were stained with DAPI (Hoechst 33342, blue) (Scale bar, 100 μm). (**C**) Quantification results of Edu-positive cells were normalized as % control. The data presented here represent the mean ± SD of at least three separate experiments. * *p* < 0.05, ** *p* < 0.01, and *** *p* < 0.001 when compared to the corresponding control group.

First, after exposure to BPAF or BPA for 24 h, the cell viability of ReNcell CX cells was measured using the CCK8 kit. The results demonstrated that to compare with the BPA, BPAF significantly reduced the cell viability of ReNcell CX cells (*p* < 0.05) (Figure 1A). Concentrations of 2 μM, 4 μM, and 8 μM of BPAF or 0.2 mM, 0.4 mM, and 0.8 mM of BPA were selected for subsequent experiments. Cytotoxicity profiling demonstrated that 0.4 mM of BPA exposure produced comparable viability reductions (78.3%) to the 4 μM of BPAF treatment (82.8%). Therefore, in subsequent experiments, not all chemicals were used at the same concentrations.

When evaluating the proliferation ability of the cells after treatment with different concentrations and using the EdU labeling method, we discovered that the number of EdU+ cells in the BPAF group decreased in a dose-dependent manner (*p* < 0.05). Similarly to the result of cell viability, the number of EdU+ cells decreased more significantly in the BPAF groups (Figure 1B,C). These results implied that, in contrast to BPA, ReNcell CX cells are more sensitive to the damaging effect of BPAF.

### 2.2. BPAF Impaired the Differentiation Direction of ReNcell CX Cells and Synaptic Growth

**Figure 2:**
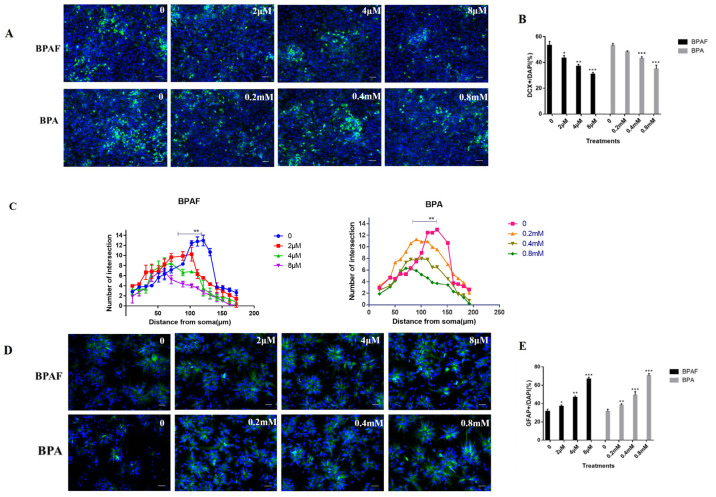
BPAF reduced the number of neurons and injured synaptic growth. (**A**) Representative immunofluorescence images of ReNcell CX cells stained with the newborn neuronal marker doublecortin (DCX, Green), while the nuclear stain was DAPI (Hoechst 33342, blue) (Scale bar, 200 μm). (**C**) Sholl analysis of the number of intersections of radial distance from the cell body (N = 30 cells). (**D**) Representative immunofluorescence images of ReNcell CX cells stained with the astrocytes marker (GFAP), and the nuclei were counterstained by DAPI (blue) (Scale bar, 200 μm). (**B**,**E**) The percentage of immunofluorescence-positive cells. One-way ANOVA was applied to test the difference between two or more groups; two-way ANOVA and Bonferroni post-test were applied for the results with Sholl analysis. The data presented here represent the mean ± SD of at least three separate experiments. * *p* < 0.05, ** *p* < 0.01, and *** *p* < 0.001 when compared to the corresponding control group.

After 24 h of exposure to BPAF, ReNcell CX cells were cultured in differentiation medium (90% basic medium +10% FBS) and changed every other day. After 5 days, immunofluorescence staining (DCX+ (doublecortin) neurons, GFAP+ (glial fibrillary acidic protein) astrocytes) was conducted. Compared with those in the BPA groups, the number of DCX+ neurons in the BPAF group decreased significantly in a dose-dependent manner (Figure 2A,B, *p* < 0.05). Interestingly, the number of GFAP+ astrocytes increased in a similar manner between BPAF and BPA treatments (Figure 2D,E, *p* < 0.05). By magnifying DCX+ cells under high magnification and counting their protrusion lengths using Image J (ImageJ 1.53k), it was found that compared with the control group, in the 2 μM group, BPAF led to a decrease in the number of neuron branches, and the length of nerve synaptic branches from the cell body was shortened, indicating that BPAF caused a reduction in the density of neuron processes and a shortening in length (Figure 2C, *p* < 0.01). Compared with BPAF-treated groups, the length of neuron processes in the BPA-treated group shortened more significantly in the 0.2 mM group and was similar to that in the BPAF-treated group in the 4 μM and 8 μM groups. The above results suggest that although BPA and BPAF are both bisphenol compounds, they cause different damages to the complexity of neurons.

### 2.3. A Total of 2 μM of BPAF Compromised the Migration Ability of ReNcell CX Cells During Differentiation

**Figure 3:**
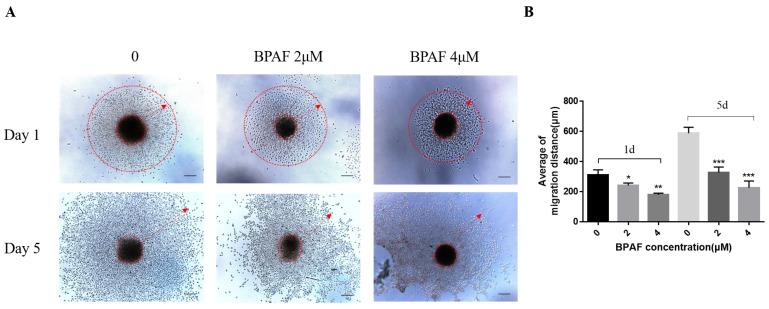
BPAF impaired the migration ability of ReNcell CX cells in the Neurosphere model. (**A**) The image of cells in the selected neurosphere which were spreading outwards, after BPAF treatment of 24 h (Scale bar, 100 μm; red arrow: the farthest distance at which cells migrated outward). (**B**) The migration average distance of cells at 1 and 5 days of differentiation were calculated by Image J software (ImageJ 1.53k). The data presented here represent the mean ± SD of at least three separate experiments. * *p* < 0.05, ** *p* < 0.01, and *** *p* < 0.001 when compared to the corresponding control group.

Based on the findings from the previous two sections, we have established that BPAF exerts significantly greater damaging effects on neural stem cells than BPA. Consequently, BPA was excluded from further experimental analysis. ReNcell CX cells were suspended and cultivated in un-coated cell culture dishes until the diameter of the neurospheres was approximately 300 μm, and neurospheres of similar sizes were planted in a pre-coated 12-well plate. The differentiation medium was replaced after 24 h of exposure to BPAF. The results indicated that the average outward migration distance of the neurospheres in the normal group was approximately 332.5 μm after differentiation for one day. In the BPAF-exposed group, the average migration distance of the 2 μM group was approximately 256.7 μm, and that of the high-dose group was approximately 182.3 μm. The difference was statistically significant (*p* < 0.05); on the 5th day of differentiation, the migration distance of the control group reached 643.7 μm, the migration distance of the 2 μM group was approximately 378.4 μm, and the migration distance of the 4 μM group was only 285.6 μm. Additionally, it could be observed that the neurites of migrating cells in the BPAF-exposed groups were shortened, which was consistent with the manner of adherent-ReNcell CX cells (Figure 3A,B). These results demonstrated that BPAF caused significant damage to the migration ability of nerve cells.

### 2.4. BPAF Increased ROS Levels in ReNcell CX Cells

**Figure 4:**
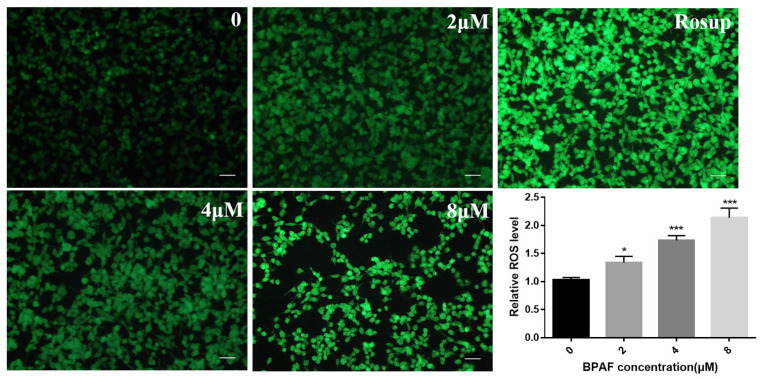
BPAF increased ROS levels in ReNcell CX cells. Immunofluorescence image of cellular ROS, ROS detected by DCFH-DA (Scale bar, 200 μm), and quantified DCF fluorescence (% of control) of ReNcell CX cells as shown by corresponding bar diagrams. The data presented here represent the mean ± SD of at least three separate experiments. * *p* < 0.05, and *** *p* < 0.001 when compared to the corresponding control group.

To further investigate the mechanism by which BPAF induces cytotoxicity in ReNcell CX cells, we measured intracellular ROS levels after exposure to BPAF. The ROS level within the cells was detected using a DCFH-DA (2′,7′-Dichlorodihydrofluorescein diacetate) reactive oxygen species probe. After exposure, DCFH-DA staining was performed and the fluorescence intensity was observed under the microscope. It was found that the intracellular ROS level increased significantly, and the fluorescence intensity increased in a dose-dependent manner (Figure 4, *p* < 0.05). Increased ROS levels may cause damage to cell structure. The elevated results indicated that BPAF might damage the development of neural stem cells. The toxicity is related to the imbalance of intracellular ROS levels.

### 2.5. Relationship Between AKT/Nrf2/HO-1 Pathway and Neurodevelopmental Toxicity of BPAF

**Figure 5:**
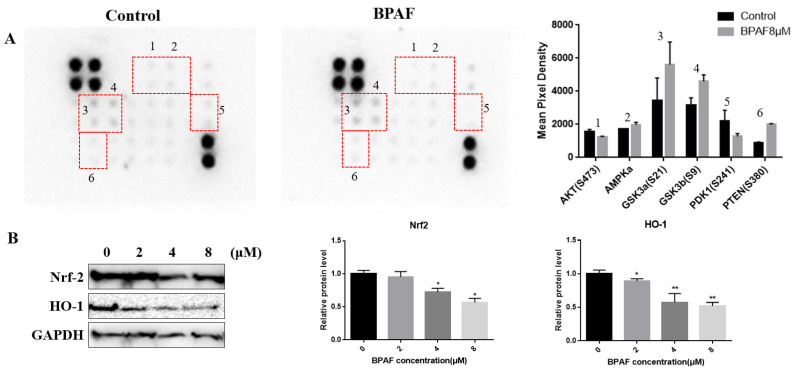
BPAF impacted neurodevelopment of ReNcell CX cells via the Nrf/HO-1 pathway. (**A**) The expression levels of AKT-related cytokines after BPAF exposure (1: AKT(S473); 2: AMPKa; 3: GSK3a(S21); 4: GSK3b(S9); 5:PDK1(S241); 6: PTEN(S380)). Total protein was collected after cells were treated with BPAF in amounts under 0, 2, 4 and 8 μM, respectively. The expression level of the detected plots collected by ImageJ (ImageJ 1.53k). (**B**) The protein expression levels of Nrf2 and HO-1 in ReNcell CX cells by Western blot. The relative quantification analysis of each protein. GAPDH was used to normalize the Western blots. The results are expressed as fold changes compared with the control. The data presented here represent the mean ± SD of at least three separate experiments. * *p* < 0.05, ** *p* < 0.01, when compared to the corresponding control group.

As an important antioxidant signaling pathway, the Nrf-2/HO-1 signaling pathway can protect the body against oxidative damage by regulating the expression of downstream antioxidant factors such as SOD (Superoxide dismutase), CAT (Catalase), and GSH (glutathione), etc. The protein expression of Nrf2 and HO-1 indicated that the expression of Nrf2 decreased in a dose-dependent manner with increasing BPAF concentrations, and the expression of HO-1 followed the same trend as that of Nrf2 (Figure 5B, *p* < 0,05). AKT requires PI3K to mediate the generation of PIP3, leading to Akt aggregation on the cell membrane. After the activation of the Akt phosphorylation pathway, which is initiated by phosphoinosite-dependent kinase 1 (PDK-1), the activation of the PI3K/Akt pathway promotes HO-1 expression and plays an anti-apoptotic role in oxidative damage responses. Therefore, we detected the expression of upstream and downstream AKT factors through AKT sequence cytokine array, as detailed in Table 1. The results are as shown in Figure 5A: After exposure to BPAF, the expression of PDK1 decreased and PTEN increased significantly, which might suggest that the PI3K/Akt pathway was inhibited in BPAF-induced developmental toxicity.

### 2.6. BPAF Altered Gene Expression Associated with the Nrf2/HO-1 Pathway

**Figure 6:**
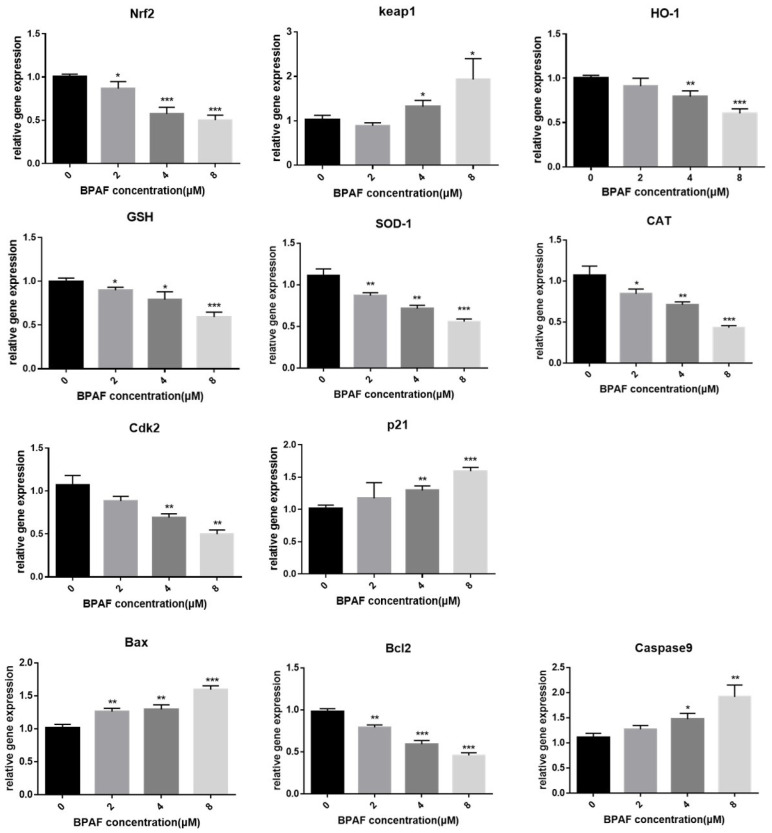
BPAF reduced the mRNA level of Nrf2/HO-1 pathway genes’ expression by qRT-PCR. The results are expressed as fold changes compared with the control. The data presented here represent the mean ± SD of at least three separate experiments. * *p* < 0.05, ** *p* < 0.01, *** *p* < 0.001 vs. the control group.

In order to further investigate the potential signaling mechanism of BPAF inducing cytotoxicity in ReNcell CX cells, we used qRT-PCR to analyze the expression of BPAF in ReNcell CX cells of genes downstream from the Nrf2/HO-1 pathway, such as *SOD-1*, *CAT*, and *GSH*, as well as the expression of genes related to cell growth and apoptosis such as *cyclin-D1*, *Cdk2* (*cyclin-dependent kinase 2*), *Bax* (*BCL-2-associated X protein*), and *Bcl2* (*B-cell lymphoma-2*). First, the mRNA expression of Nrf2 and HO-1 was consistent with their protein expression after BPAF treatment, whereas the mRNA expression of downstream antioxidant factors decreased in a dose-dependent manner (Figure 6). In addition, both *Cdk2* and *p21* are associated with G1 phase arrest. After BPAF treatment, the mRNA level of *p21* increased and that of *Cdk2* decreased (both *p* < 0.05, Figure 6), suggesting that there may be DNA damage. Changes in the expression of *Bax*, *Bcl2* and *Caspase9* (*p* < 0.05) also showed an increase in caspase9-dependent apoptosis after BPAF was impaired.

## 3. Discussion

Over the past few decades, it has been demonstrated that BPA can induce various reproductive, somatic, and developmental defects. Many countries have taken the lead in prohibiting the use of BPA in certain consumer products. However, many companies use some BPA substitutes to replace BPA in products labeled “BPA-free” [21]. Current research has revealed that some BPA analogs can cause developmental defects. There have also been reports in some animal models indicating that certain analogs, including BPAF, are more toxic and estrogenic than BPA itself [22]. Some studies have demonstrated that male adolescents exhibited peak serum and gonadal concentrations (Cmax) of 27.7 pM for BPAF and 135 pM for BPA. Comparatively, BPAF showed enhanced glucuronidation efficiency versus other bisphenols within the concentration spectrum observed in hepatic and intestinal tissues. Notably, BPAF appears to circumvent first-pass intestinal metabolism, achieving direct entry into systemic circulation. This metabolic bypass mechanism resulted in cerebral concentrations reaching 2.04-fold of the corresponding serum level [23]. In this study, for the first time, we selected a kind of neural progenitor cell line (ReNcell) derived from the cortical area of human fetal brain tissue as a research model. Based on the previous literature and preliminary experiments, we determined that the doses of BPAF and BPA were 2, 4, and 8 μM (BPAF) and 0.2 mM, 0.4 mM, and 0.8 mM (BPA), respectively. We found that BPAF significantly damaged ReNcell CX cells at lower doses and this damaging effect may be related to the Nrf2/HO-1 pathway.

Developmental neurotoxins are a group of substances that may disrupt the normal development of the nervous system. If not intervened promptly, they may adversely impact the normal development of the structure and/or function of the nervous system. Although there are numerous potential targets for neurotoxins, the final biological effects are often manifested in one or more key developmental nodes (such as proliferation, differentiation, migration, synaptic growth, etc.) [24,25]. In this study, we examined the damaging effects of BPAF and BPA during the proliferation, differentiation, and migration of ReNcell CX cells and found that BPAF is more cytotoxic than BPA in terms of influencing proliferation, which is similar to the research trend of BPAF in zebrafish neurotoxicity [26]. The differentiation of ReNcell CX cells into specific functional cells is crucial for the development of the nervous system. Studies have indicated that after administration of BPAF and BPA, BPAF treatment adversely affects the axon length of motoneurons in zebrafish and reduces its central nervous system (CNS) neurogenesis [27]. Our experimental findings also indicated that exposure to BPAF and BPA could interfere with the differentiation direction of ReNcell CX cells, decrease the number of neurons generated, and increase the differentiation into astrocytes. Nevertheless, BPAF reduced the number of neurons more significantly. The examining of neurite growth revealed that the decrease in neurite density and the shortening of neurite length are more pronounced in the BPAF group. In addition, the ability of differentiated neurons to migrate to specific areas is also a crucial part in assessing the development of the nervous system. We employed a neurosphere model to simulate the migration process in vitro and found that even in the BPAF (2 μM) group without obvious cytotoxicity, the ability of neuron cells to migrate outward was significantly reduced 5 days after differentiation, and abnormal neuron migration may lead to the development of a certain brain structure. Given the general increase in BPAF exposure and the particularity of nervous system development, the safety of BPAF demands should be urgently attended to.

The nervous system is particularly prone to oxidative damage because of its high metabolic rate, generation of hyperoxygenated metabolites, relatively low levels of antioxidants, poor repair capabilities, and a high lipid composition that is vulnerable to peroxidation and oxidative modification by ROS [28]. The Nrf2/HO-1 pathway plays a crucial role in resisting damage caused by oxidative stress. Studies have indicated that oxidative stress and apoptosis caused by BPAF may translate into phenotypic behavior and neurodevelopment abnormalities in zebrafish [29]. Some studies have shown that the Nrf2/HO-1 signaling axis plays a key role in the process of PD, and there is often a decrease in the expression of Nrf2 and HO-1 in certain nervous system diseases [30]. In this study, we found that ROS levels significantly increased with increasing concentration after exposure to BPAF, and the expressions of Nrf2, HO-1, and their downstream antioxidant factors such as SOD, CAT, and GSH were markedly decreased, which was consistent with the results in the existing literature, indicating that the Nrf2/HO-1 pathway might be one of the mechanisms by which BPAF impairs neural development. Studies have shown that ROS could damage cell growth and development by causing DNA damage and cell cycle arrest [31]. This study detected that P21 expression increased and that of CDK2 decreased after exposure to BPAF, which also indicated the presence of cell cycle arrest. In addition, some studies have shown that the Nrf2 is one of the target proteins of AKT [32]. Considering the significant role of Nrf2 in resisting oxidative stress induced by exogenous factors, we employed AKT gene chip analysis and found that in the BPAF-treated group, there was a notable decrease in the AKT activator PDK1. PDK1 can activate Akt and subsequently participate in the activation of the phosphatidylinositol 3 kinase (PI3K)-Akt signaling pathway, indicating that there was an inhibition of AKT activation during the process of neural stem cells damaged by BPAF. Although the expression of AKT (ser473) has not been significantly reduced, a study confirmed that ROS could downregulate the expression of PDK1 (the PDH kinase), which resulted in a less phospho-PDH phenotype which could interact with Keap1, the negative controller of NRF2, directly leading to NRF2 decrease [33]. In the future, we will continue to explore whether the regulation of the phospho-PDH phenotype is involved in BPAF inducing ReNcell CX cell development through the Nrf2/HO-1 pathway in subsequent experiments.

In summary, exposure to BPAF can induce abnormal development of neural stem cells, reduce their proliferation, inhibit their differentiation into neurons, and damage the migration ability of neural cells. The damage mechanism might be related to oxidative stress induced by BPAF through the Nrf2/HO-1 pathway. Nevertheless, there are still numerous shortcomings in this study, and how BPAF inhibits the Nrf2/HO-1 pathway and its possible intervention targets require further investigation.

## 4. Materials and Methods

### 4.1. Cell Culture and Treatments

The ReNcell CX cell line (Merck Millipore, Darmstadt, Germany; Cat. No. SCC007) was identified as immortalized human neural progenitor cells, derived from the cortical region of human fetal brain tissue. After adding the diluted laminin solution (20 µg/mL) to cover the whole surface of the tissue culture-ware 4 h, ReNcell CX cells were maintained at 37 °C with 5% CO_2_ in ReNcell NSC Maintenance Medium (Millipore Cat. No. SCM005, Burlington, MA, USA) supplemented with 20 ng/mL of FGF-2 and 20 ng/mL of EGF (PeproTech, Rockville, NJ, USA). In the neurosphere model, ReNcell CX cells were maintained in suspension culture until the neurosphere diameter reached approximately 100 μm for subsequent experimental studies. The number of passages for all experimental cells should be controlled within the range of 5 to 30 generations. In our study, cells were subcultured until passage 7 for experiments.

Bisphenol A (BPA; ≥99% purity, Sigma-Aldrich, Saint Louis, MO, USA) and Bisphenol AF (BPAF; ≥99% purity, Sigma-Aldrich, Saint Louis, MO, USA) were each prepared as stock solutions in dimethyl sulfoxide (DMSO) and stored at −20 °C. Serial dilutions of the stock solutions were prepared in complete medium prior to application, with the final DMSO concentration in application solutions maintained below 0.01% (*v*/*v*). BPAF (0 μM, 2 μM, 4 μM, and 8 μM) or BPA (0.2 mM, 0.4 mM, and 0.8 mM) were treated for 24 h when the cells were over approximately 80% confluent. Three replicates were designed for each dose.

### 4.2. Cell Activity and Proliferation Measurement

Cell viability was measured by using a colorimetric cell counting kit (CCK-8, Dojindo, Mashiki, Japan). Cells were seeded in 96-well plates at 1 × 10^4^ cells/well density. Following overnight incubation at 37 °C with 5% CO_2_, ReNcell CX cells were exposed to various concentrations of BPAF (0 μM, 2 μM, 4 μM, 8 μM, 16 μM, 32 μM, 64 μM) or BPA (0 mM, 0.2 mM, 0.4 mM, 0.8 mM, 1.6 mM, 3.2 mM) for 24 h. (The treatment concentrations were chosen by published plasma concentrations of BPAF and BPA in pregnant populations (1.7 nM and 65.7 nM, respectively) [34], and their corresponding IC50 values for cytotoxicity in ReNcell neural progenitor cells.) At the end of the treatments, the medium was subsequently discarded, and 110 μL of new medium containing CCK-8 solution (10 μL) was added to each well and incubated at 37 °C for 4 h. Absorbance was measured at 490 nm using a SYNERGY Microplate Reader (BioTek, Winooski, VT, USA).

Cell proliferation was measured using the 5-ethyl-2′-deoxyuridine incorporation (EdU) method [35]. Specifically, after the cells in the 96-well plates were treated with BPAF or BPA, 10 μM of EdU was added to the flash medium for incubation for 2 h, and then fixed with 4% paraformaldehyde for 15 min at room temperature. Staining observations were carried out using Cell-Light TM EdU Apollo 488 Kit (Ribobio, Guangzhou, China) in accordance with the test protocol. Images were acquired using an Olympus microscope system.

### 4.3. Immunocytochemistry

The differentiation fate of was analyzed by utilizing immunocytochemistry. When confluence reached 80%, they were seeded into a laminin pre-coated 24-well slide at a density of 3 × 10^5^ cells/well and cultured overnight before exposure to the designated treatments. The immunocytochemistry was performed as previously described [36]. The primary and secondary antibodies are anti-rabbit DCX, anti-mouse GFAP (1:200, Cell Signaling Technology, Danvers, MA, USA), and Alexa Fluor GFAP-labeled goat anti-rabbit antibody (1:200, Beyotime, Haimen, China), respectively. Nuclei were counterstained with DAPI staining solution (1:10,000, Beyotime, Haimen, China). Fluorescence images were obtained by using an inverted fluorescent microscope with a 10× objective lens. The counted cells in each group were captured in five fields, and the percentage of DCX or GFAP-positive cells was calculated by Image J (ImageJ 1.53k). The Neurite outgrowth of neurons was examined by the Sholl analysis plugin of Image J (ImageJ 1.53k), which evaluated the intersections of the radial distance of 30 DCX-positive cells from the cell body.

### 4.4. Intracellular ROS Levels

After treatment with BPAF and the positive agent H_2_O_2_, the cells were washed with PBS. All the samples were stained with the DCFH-DA probe (Beyotime, Haimen, China), incubated at 37 °C for 20 min, and washed 3 times with PBS to ensure complete removal of the probe. The ROS concentration was measured at λex/em = 488/525 nm. The fluorescence intensity was analyzed using a SpectraMax multimode microplate reader (Tecan, Hombrechtikon, Switzerland).

### 4.5. Proteome Profiling Array

RayBio^®^ human cytokine array C series (RayBiotech, Inc., Norcross, GA, USA) was used to detect the expression levels of cytokines after treatment with BPAF. After treatment for 24 h, the total protein of each group was extracted and the content was determined using BCA Assay Kit (Bicinchoninic Acid protein Quantification kit, Sigma-Aldrich, Saint Louis, MO, USA). 200 µg protein sample was placed on an array membrane and incubated overnight at 4 °C. Bound cytokines were tested in accordance with the instructions and finally examined using a Doc Molecular Imager (Bio-Rad, Hercules, CA, USA). The area under the curve (AUC) was quantified by ImageJ (ImageJ 1.53k).

### 4.6. Western Blot

After treatment with BPAF (0, 2, 4, and 8 μM), cells were washed twice with PBS and then lysed in RIPA lysis buffer for 10 min. Subsequently, all the samples were centrifuged at 12,000 rpm for 5 min at 4 °C. The specific protein concentration of the supernatant was determined by the BCA method. After all protein samples were fixed in available loading buffer and boiled for 5 min, equal amounts of protein (20 µg protein/well) were subjected to SDS polyacrylamide gel electrophoresis (10%), then transferred to a PVDF membrane (Millipore, Burlington, MA, USA) and incubated overnight at 4 °C with diluted primary antibodies of Nrf-2, HO-1 and GAPDH. After incubation, the samples were washed 3 times with 1× TBS-T for 15 min, and then incubated with enzyme-linked secondary antibody for 1 h. Bands were detected using an ECL substrate (Thermo Scientific, Waltham, MA, USA) and Doc Molecular Imager (Bio-Rad, USA). GAPDH was used as the reference protein, and the full antibody was obtained from Santa Cruz Biotechnology, Dallas, TX, USA. The area under the curve (AUC) was calculated from ImageJ (ver.; 15.4 b, NIH, USA) quantitative bands. For the Western blot analysis, after being transferred to the PVDF membrane and blocking, the membranes were cut prior to hybridization with primary antibodies. The cut size of membranes followed the prestained protein marker (Bio-Rad, Hercules, CA, USA). For Nrf-2 (97–100 kDa), the cut size was above 50 kDa; the HO-1 (28 kDa) was below 37 kDa and the GAPDH (37 kDa) was below 75 kDa. The final images were captured with Doc Molecular Imager (Bio-Rad, Hercules, CA, USA) from 30 to 300 s.

### 4.7. Quantitative Real-Time PCR Analysis

Total RNA was extracted from treated ReNcell CX cells using the TaKaRa MiniBEST Universal RNA Extraction Kit (TaKaRa, Osaka, Japan) and the concentrations were determined using NanoDrop 2000 (Thermo Scientific, Waltham, MA, USA). For the synthesis of the first-strand cDNA, 2 µg of total RNA was incubated at 65 °C for 5 min to dissolve the secondary structures. Subsequently, the ReverTra AceTM qPCR RT Master Mix (TOYOBO, Osaka, Japan) was employed for annealing at 37 °C, 50 °C, and 98 °C for 15 min, 5 min, and 5 min, respectively. cDNA (100 ng) was utilized as a quantitative reverse transcription PCR (RT-qPCR) template with QuantStudio 7 Flex (Applied Biosystems, Waltham, MA, USA) and PowerUp™ SYBR™Green Master Mixes (Applied Biosystems, Waltham, MA, USA). The qRT-PCR program is as follows: 50 °C, 2 min; 95 °C, 2 min; 95 °C, 15 s, 60 °C, 1 min, 30 cycles. The procedure was completed at 95 °C for 15 s, 60 °C for 1 min, and 95 °C for 15 s to verify the specificity of the amplification products. All RT-qPCR data were evaluated using the 2^−ΔΔCt^ method. β-actin was used as a reference gene. The primer sequences used in this study can be found in Table 2. All raw Ct values could be found in Supplementary Materials.

### 4.8. Statistical Analysis

All the experiments were repeated at least 3 times. Statistical analysis was performed using SPSS 22.0. One-way ANOVA was used to test the difference between two or more groups. The results of three independent experiments were expressed as the means ± SEMs. *p* < 0.05 indicated that the difference is statistically significant.

### 4.9. Ethics and Provenance Statement

The ReNcell CX was developed by the ReNeuron Group plc, a biotech company that specializes in using human somatic stem cells for therapeutics. The cells have been obtained in a legal and ethical manner, compliant with current local informed consent procedures.

## 5. Conclusions

Using human neural stem cells cultured in vitro as a model, exposure to BPAF can induce abnormal development of neural stem cells, decrease proliferation, and inhibit their differentiation into neurons and the migration ability of damaged neural cells. The damage mechanism might be related to oxidative stress induced by BPAF via the Nrf2/HO-1 pathway.

### Study Limitations

In this study, two crucial limitations should be noticed. One limitation is the use of ReNcell CX cells. Although this cell line is a single immortalized neural progenitor cell line and has been wildly used in related studies, it cannot fully represent the real situation in human body. Another limitation is that the mechanistic findings should be replicated in either primary human NSCs or organoids to improve the generalizability and translational values. In future studies, we intend to employ advanced models such as organoids, while also implementing knockout or overexpression of key proteins within the Nrf2/HO-1/AKT pathway to comprehensively validate the role of this pathway in BPAF-induced neurotoxicity.

## Figures and Tables

**Table 1:** Antibody Array Map (Each antibody is spotted in duplicate vertically).

A	B	C	D	E	F	G	H
POS	POS	NEG	NEG	AKT(S473)	AMPKa	BAD(S112)	4E-BP1(T36)
POS	POS	NEG	NEG	AKT(S473)	AMPKa	BAD(S112)	4E-BP1(T36)
IC	GSK3a(S21)	GSK3b(S9)	mTOR(S2448)	p27(T198)	IC	P70S6K(T421/S424)	PDK1(S241)
IC	GSK3a(S21)	GSK3b(S9)	mTOR(S2448)	p27(T198)	IC	P70S6K(T421/S425)	PDK1(S242)
PRAS(S40)	PTEN(S380)	RAF-1(S301)	RPS6(S235/236)	IC	IC	NEG	POS
PRAS(S41)	PTEN(S381)	RAF-1(S302)	RPS6(S235/237)	IC	IC	NEG	POS

**Table 2:** Primers used in qRT-PCR.

Primer Name	Forward (5′-3′)	Reverse (3′-5′)
*Nrf2*	ACGGTATGCAACAGGACATTGAGC	TTGGCTTCTGGACTTGGAACCATG
*Keap1*	ATTCAGCTGAGTGTTACTACCC	CAGCATAGATACAGTTGTGCAG
*HO-1*	CCTCCCTGTACCACATCTATGT	GCTCTTCTGGGAAGTAGACAG
*SOD*	ATCCTCTATCCAGAAAACACGG	GCGTTTCCTGTCTTTGTACTTT
*GSH-Px*	GTTGCCTGGAACTTTGAGAAG	CTCGATGTCAATGGTCTGGAAG
*CAT*	GAGCACAGCATCCAATATTCTG	CTCATTCAGCACGTTCACATAG
*Bax*	AGGATGCGTCCACCAAGAAGCT	TCCGTGTCCACGTCAGCAATCA
*Bcl2*	CCTGTGGATGACTGAGTACCTG	AGCCAGGAGAAATCAAACAGAGG
*Caspase9*	CTGCTGCGTGGTGGTCATTCTC	CACAATCTTCTCGACCGACACAGG
*Cdk2*	CGCACCTTTCAGACCGCTGTTT	CCATCTCCTCTATGACAGC
*P21*	TCGCTGTCTTGCACTCTGGTGT	CCAATCTGCGCTTGGAGTGATAG
*β* *-actin*	CATTGCTGACAGGATGCAGAAGG	TGCTGGAAGGTGGACAGTGAGG

Note: Primer sequences were all from OriGene Technology (Beijing, China).

## Data Availability

All other relevant data supporting the key findings of this study are available within this article.

## Supplementary Materials

The following supporting information can be downloaded at: https://www.mdpi.com/article/10.3390/ijms26125685/s1.
